# Psychosocial characteristics differentiate non-distressing and distressing voices in 10,346 adolescents

**DOI:** 10.1007/s00787-019-01292-x

**Published:** 2019-02-28

**Authors:** Else-Marie Løberg, Rolf Gjestad, Maj-Britt Posserud, Kristiina Kompus, Astri J. Lundervold

**Affiliations:** 1 Department of Addiction Medicine, Haukeland University Hospital, Bergen, Norway; 2 Division of Psychiatry, Haukeland University Hospital, Bergen, Norway; 3 Department of Clinical Psychology, University of Bergen, Bergen, Norway; 4 NORMENT Center of Excellence, Haukeland University Hospital, Bergen, Norway; 5 Centre for Research and Education in Forensic Psychiatry, Haukeland University Hospital, Bergen, Norway; 6 Department of Clinical Medicine, University of Bergen, Bergen, Norway; 7 Department of Biological and Medical Psychology, University of Bergen, Bergen, Norway; 8 School of Natural Sciences and Health, Tallinn University, Tallinn, Estonia; 9 Gillberg Neuropsychiatry Centre (GNC), University of Gothenburg, Gothenburg, Sweden; 10 K. G. Jebsen Center for Neuropsychiatric Disorders, University of Bergen, Bergen, Norway

**Keywords:** Verbal auditory hallucination, Psychosis, Adolescent psychiatry, Early intervention, Risk factors

## Abstract

Adolescents hearing non-existent voices may be at risk for psychosis, but the prevalence of voice-hearing (VH) in the general population complicates clinical interpretations. Differentiating between VH with and without distress may aid treatment decisions in psychosis services, but understanding the differences between these two phenomena as they present in the normal adolescent population is necessary to validate this differentiation. The present study compared VH with and without distress in 10,346 adolescents in relation to clinical characteristics, known risk factors, predictors and psychosocial moderators of psychosis. A population-based cohort of Norwegian 16–19 years old adolescents completed a comprehensive web-based questionnaire, including two questions from the extended Launay-Slade Hallucinations Scale: (1) I often hear a voice speaking my thoughts aloud and (2) I have been troubled by hearing voices in my head. Adolescents reporting no VH, non-distressing VH or distressing VH were compared on 14 psychosocial and clinical variables. A multinomial regression model showed that non-disturbing voices were predicted by better school grades, social dysfunction, distractibility, affective symptoms and experience of trauma, while the disturbing voices were predicted by the experience of bullying and trauma, perceived negative self-worth and self-efficacy, less family support, dysregulation of activation, distractibility, self-harm and anxiety. Hearing voices without distress versus being distressed by the voices is related to different constellations of psychosocial variables, suggesting that they represent two separate groups of adolescents. The findings validate the emphasis on distress in clinical practice.

## Introduction

Hearing non-existent voices is a potential marker of psychosis development, and may create subjective distress and anxiety with corresponding functional decline and mental health problems. However, voice-hearing (VH) is quite common in the age period when psychosis develops, complicating clinical interpretation [1]. The prevalence of VH and auditory hallucinations (AVH) in the general population has been estimated to be around 4–7.5% [2–5]. Importantly, there seems to be a developmental effect, with psychotic symptoms (predominantly AVH) occurring in 17% of children aged 9–12 but only in 7.5% of adolescents aged 13–18 years [6]. A previous study from the present dataset found that 10.6% of a population-based sample of 9646 adolescents reported often hearing a voice speaking thoughts out loud [7]. However, only 5.3% reported that they experienced being troubled by voices, suggesting two groups of VH adolescents; a relatively large group hearing voices without feeling distressed, and a smaller group being distressed by the voices.

Both these groups may be referred to early psychosis intervention services, but the existence of distress may guide further treatment decisions [8], since distress may predict transition to psychosis [9]. Distress during VH, related to the existence of a non-realistic, psychotic interpretation of the voices that consequently increases anxiety, vigilance and personal suffering, is of clinical importance. VH may be considered to be several different phenomena: a symptom of psychosis [10], a symptom of psychosis risk; often depicted as “high-risk” (HR) “ultra-high risk”, “prodromal state” and “at-risk mental state” for psychosis, as well as a “psychosis-(like) experience” (PLE/PE), or as a normal phenomenon [8, 11–13]. Not being distressed suggests a non-psychotic interpretation; e.g. “I am hearing voices because I am tired” or “Sometimes my mind plays tricks on me” (for details, see [14, 15]). However, one may question the emphasis on distress, as there is limited knowledge on whether non-distressed voice-hearers are different to voice-hearers who are distressed by their voices. Do these groups differ in psychosocial characteristics known to be related to the development of psychosis? Research on adults demonstrates that there is a considerable high number of voice-hearers in the population who do not need psychiatric care [16, 17]. By examining characteristics of different voice-hearers in early development, more light could be shed on the possible symptom trajectories of voice-hearing individuals. If the non-distressing VH experience is a variation of normal perceptual phenomena, while the distressed adolescents are on a (non-deterministic) pathway to psychosis, the latter group should possibly have more psychosis-related characteristics, typically; (1) comorbid mental health symptoms (2) risk factors for psychosis (3) predictors of psychosis conversion, and (4) fewer psychosocial moderators and resilience factors.

Several mental health conditions often co-exist with psychosis. The most prevalent are affective symptoms [18], anxiety [19] and illicit substance abuse [20–26]. General markers of sub-optimal mental health are typically present before psychosis onset, such as anxiety and depressive disorders [11] and dysregulated activation [27]. Self-harm is particularly prevalent in early untreated psychosis phases [28], and is an important marker of mental distress in adolescents. Psychosocial variables may alone or in interaction with other variables or genetic vulnerability heighten the risk for psychosis [29], e.g. illicit substance use [30]. Cannabis has been implicated in psychosis development in prospective studies [31, 32], especially for moderate to severe use and/or if started in the early teens [33–35]. Similarly, other substances acting on dopamine receptors have been linked to psychosis development, especially (meta)amphetamines [36]. There is also an increasing international consensus that childhood trauma is a risk factor for psychosis [37–41] and for PEs [42], with reports of an almost three–fold greater likelihood of experiencing psychotic symptoms after childhood trauma [43], with a dose–response relationship [39, 40, 43–46]. A dose–response relationship of a history of bullying and psychotic experiences has also been reported [47].

Predictors of conversion to psychosis include attenuated positive symptoms and functional decline in areas of school and social functioning. General and social functioning has been shown to be significantly lower for child and adolescent patients with psychotic-like experiences than without [48, 49]. Negative symptoms and social withdrawal [50] predict psychosis, typically seen as reduced school attendance in youngsters. In a five year prospective study conversion to psychosis was best predicted by disorganized communication, suspiciousness, verbal memory deficits, and decline in social functioning [51]. Furthermore, cognitive deficits [52, 53] and being part of high-risk populations [54, 55] are shown to be predictive of psychosis conversion [56–58]. For adolescents, cognitive difficulties are typically reflected by lower school grades.

Finally, there may be moderators of psychosis risk related to psychological resilience; family support, self-efficacy and experience of self-worth. Smith and colleagues [59] found that lower self-esteem was related to severity and negative content of AVH, and negative self-evaluations have been shown to be strongly related to psychosis even when depression has been controlled for [60, 61]. Reduced self-efficacy—an individual's belief in his or her capacity to execute behaviors necessary to produce specific performance attainments [62]—has been shown also in patients at-risk for psychosis [63]. Family support is important for avoiding new episodes of psychosis, [60, 64, 65], but is decreased and related to symptomatic and functional improvement in HR patients as well [49, 66].

By including information regarding the risk factors and characteristics described above, the present study aims to test the validity of a distress-based distinction of the concept of VH. We expect to find a more psychosis-associated psychosocial profile among the distressed adolescents compared to the voice-hearers not distressed by the voices; more comorbid mental health symptoms (anxiety, affective symptoms, dysregulation of activation, self-harm), risk factors for psychosis (experience of trauma, bullying, illicit drug use) and predictors of psychosis conversion and markers of mental distress (social functioning, school grades, days absent from school, distractibility) and fewer psychosocial moderators and resilience factors (family support, self-efficacy, experience of self-worth).

## Methods

### Sample

The present study included a population-based sample of adolescents participating in the fourth wave of the Bergen Child study (see https://uni.no/en/uni-health/rkbu-vest/the-bergen-child-study/ for details), headed by the Regional Centre for Child and Youth Mental Health and Child Welfare, Norce Norwegian Research Center. The sample included all adolescents attending high school during the spring 2012, mostly students born between 1993 and 1995. The adolescents received information about the study to their homes and/or via their school e-mail, and completed the internet-based questionnaire at school upon consent. The study was approved by the Regional Committee for Medical and Health Research Ethics in Western Norway. The current study is based on the first version of quality-assured data files released in May 2012. Information about sex and year of birth were based on the personal identity number in the Norwegian National Population Register. Of the included adolescents (*n* = 19,121), 11,092 completed or filled in parts of the questionnaire. Some subjects (*n* = 746) were removed due to missing personal identity numbers and unreliable answer profiles resulting in a total sample of 10,346 adolescents in the present study. The sample has previously been found to be representative of Norwegian high school youth [67].

### Assessment

#### Voice-hearing

Presence of VH was assessed with two items from extended Launay-Slade Hallucinations Scale (LSHS-E) [68]; (1) “I often hear a voice speaking my thoughts aloud”; and (2) “I have been troubled by hearing voices in my head”. Responses are given on a 5-point scale (0 = “certainly does not apply to me”, 1 = “possibly does not apply to me”, 2 = “unsure”, 3 = “possibly applies to me” and 4 = “certainly applies to me”). Factor analyzes of the LSHS-E have shown that both of these items load on an underlying “auditory hallucinations” factor [68, 69], but in addition, item 2 load on a factor associated with psychopathology [70, 71], and has been associated with higher degree of negative emotionality than item 1 [72]. The five response categories to the two VH items were recoded to responses “0″ and “1″ being coded as “No endorsement” (0) and responses “3″ and “4″ being coded as endorsement (1). Adolescents answering “2″ were coded as unsure and were excluded from the group analysis (*n* = 473). The following three groups were examined (1) No VH (no endorsement, both items) (2) VH (endorsement of hearing voices only) and (3) Distressing VH (endorsement of distressing voices). This procedure resulted in a score in one of these three response categories only, thus the subjects were only allocated to one of the three groups.

#### Predictor variables

The predictor variables were based on selected items about psychosocial, demographic and clinical status and characteristics chosen from the web-based questionnaire based on previous studies on factors related to early psychosis, see Table 1 for details. To validate the constructs and content, seven of the predictor variables were derived using confirmatory factor analyzes (factor loadings not given, goodness of fit: *χ*^2^ = 17,665.639, *p* < 0.001, CFI =0.92, TLI =0.91, RMSEA =0.048, 90% RMSEA CI 0.047–0.049, RMSEA close fit =1.000). For the other seven predictor variables factor analysis was not required since the relevant questions were clearly defined with narrow connotations. Six of these variables were based on one single item and one was based on 4 items. The non-factor analyses variables were; (1) Experience of trauma; 4 items about negative life events from a previous Norwegian national survey [73] (2) Experience of bullying; from the national Olweus bullying survey [74] (3) Illicit drug use; developed for the web-based questionnaire [75] (4) Self-harm; from the Child and Adolescent Self-harm in Europe (CASE) Study [76] (5) Anxiety; from the Screen for Child Anxiety Related Disorders; SCARED [77] (6) School grades; mean grades from official registers from the Hordaland County, excluding Physical Education, and (7) Days absent from school (web-based questionnaire).

**Table 1:** Overview of predictor variables

Variables	Questions
Experience of trauma	Have you experienced any of the following events? A disaster or serious accident Violence from an adult, like being beaten, had your hair pulled or the like Seen or heard that someone you care about has been exposed to violence from an adult Unwanted sexual acts
Experience of bullying	How often have you been bullied at school the last months?
Illicit drug use	Have you ever tried hashish, marihuana or other narcotic substances?
Self-harm	Have you ever deliberately taken an overdose (e.g. of pills or other medication) or tried to harm yourself in some other way (such as cut yourself)?
Anxiety	I get really frightened for no reason at all
School grades	Mean grades from school records, not including gymnastics, 1-–6, 6 highest grade
Days absent from school	How many days have you been absent from school the last month?
Experience of self-worth	I felt I was no good any more I hated myself I was a bad person I felt lonely I thought nobody really loved me I thought I could never be as good as other kids I did everything wrong
Family support	I reach my goals if I work hard I am satisfied with my life until now In my family we agree on what is important in life I am happy together with my family I always have someone who can help me when I need it My family has a positive outlook even if something very sad happens In my family, we support each other In my family we like to do things together I have some close friends/family members who appreciate my personal characteristics
Distractibility	How often do you have trouble wrapping up the final details of a project, once the challenging parts have been done? How often do you have difficulty getting things in order when you have to do a task that requires organization? How often do you have difficulty keeping your attention when you are doing boring or repetitive work? How often do you have difficulty concentrating on what people say to you, even when they are speaking to you directly? I am easily distracted, I find it difficult to concentrate I finish the work I’m doing. My attention is good I found it hard to think properly or concentrate
Social functioning	Do you find it difficult to socialize with, or to get in touch with people, especially people your own age? Do you prefer to be alone rather than being together with other people? Do you have difficulties perceiving social cues? I easily find new friends I am good at talking to new people
Dysregulation of activation	How often do you feel overly active and compelled to do things, like you were driven by a motor? How often do you feel restless or fidgety? How often do you find yourself talking too much when you are in social situations? I am restless, I cannot stay still for long I am easily distracted, I find it difficult to concentrate I was very restless
Self-efficacy	I feel that I am skillful My belief in myself gets me through difficult times In adversity I tend to find something that makes me grow as a person
Affective symptoms	I felt miserable or unhappy I didn’t enjoy anything at all I felt so tired I just sat around and did nothing I felt I was no good any more I cried a lot I found it hard to think properly or concentrate I felt lonely

The factor analyses-based variables were (1) Family support; nine items from the Resilience Scale for Adolescents; READ [78]; (2) Self-efficacy; three items from READ (3) Distractibility, four items from the adult ADHD Self-Report Scale; ASRS [79], two items from The Strengths and Difficulties Questionnaire; SDQ [80] and one item from the Short Mood and Feelings Questionnaire; SMFQ [81]; (4) Dysregulation of activation; three items from ASRS, two items from SDQ and one items from SMFQ (5) Experience of self-worth; seven items from SMFQ (6) Affective symptoms; seven items from SMFQ, and (7) Social functioning; three items from the Autism symptom self-report; ASSERT [82] and two items from READ.

#### Statistics

IBM SPSS Statistics 23 was used for descriptive analyses [83]: mean, standard deviation (SD) and skewness. Factor scores were estimated through confirmatory factor analysis with the program Mplus 7.4 [84]. Since the outcome variable was nominal with three categories, a multinomial regression model was analyzed with SPSS. All predictors were included simultaneously in one block [85], and give adjusted estimates for all predictor variables. Thus, this model analyzes the effect of “hearing voices” and “being troubled by voice hearing” in contrast to no voice hearing as the reference category.

## Results

### Descriptive results

Of the 10,346 adolescents, 53.5% were girls. The mean age was 17.70 years (SD =1.60). The prevalence of VH was No VH = 80.6% (*N* = 7954), VH =13.9% (*N* = 1371), and Distressing VH = 5.6% (*N* = 548), yielding a total of *n* = 9873 (excluding adolescents answering “unsure”). The descriptive results for the predictor variables are presented in Table 2 and 3. The results indicate relatively low prevalence of endorsement in general, except for Anxiety with 15% endorsement.

**Table 2:** Frequencies of the categorical predictor variables

Variables	%
Experience of bullying	
Not bullied	91.4
Occasionally	5.0
2–3 times a month	1.1
Weekly	0.5
Several times a week	0.7
Self-harm	
No	89.5
Once	4.7
Several times	5.8
Anxiety	
Not true or hardly ever true	84.2
Somewhat true or sometimes true	12.1
Very true or often true	3.7
Illicit drug use	
Yes, have tried	11.5

**Table 3:** Means and standard deviations of the continuous predictor variables

Variables	Mean	SD
Experience of trauma	0.76	1.35
Days absent from school	4.25	5.35
School grades	3.83	0.81
Experience of self-worth	0.00	0.41
Family support	0.00	0.73
Distractibility	0.00	0.59
Social functioning	0.00	0.31
Dysregulation of activation	0.00	0.80
Self-efficacy	0.00	0.89
Affective symptoms	0.00	0.54

### Prediction of voice-hearing

The results from the predictor model of VH are presented in Table 4. The outcome variable was specified with the predictor effect on “VH” and “distressing VH” against the “No VH” as the reference category. Models for subjects between 16–19 and 16–25 years old are presented. No evidence for high multicollinearity between the predictor variables emerged, indicated by VIF with the highest value of 4.75. One predictor correlation was found to be high (*r* = 0.86), with 73% explained common variance.

**Table 4:** Results from multinomial regression for the categories “voice-hearing” and “distressed voice-hearing” in contrast to “no voice-hearing” (reference category)

	Sample: 16–19 years old					Sample: 16–25 years old				
Predictors of	*b*	*p*	Exp (B)	CI Low	CI High	*b*	*p*	Exp (B)	CI Low	CI High
Voice-hearing										
Experience of bullying	− 0.00	0.992	1.00	0.87	1.15	− 0.01	0.876	1.00	0.86	1.13
Experience of trauma*	0.08	0.002	1.09	1.03	1.15	0.06	0.019	1.06	1.01	1.12
School absence (days)	0.01	0.250	1.01	0.99	1.02	0.01	0.295	1.01	0.99	1.02
School grades*	0.21	<0.001	1.24	1.12	1.37	0.19	< 0.001	1.22	1.11	1.34
Negative Self-worth	0.22	0.189	1.24	0.90	1.71	0.19	0.226	1.21	0.89	1.64
Family support	− 0.02	0.818	0.98	0.84	1.15	− 0.01	0.930	0.99	0.86	1.15
Distractibility*	0.56	<0.001	1.74	1.43	2.13	0.53	<0.001	1.70	1.40	2.05
Social dysfunction*	0.45	0.009	1.57	1.12	2.21	0.45	0.007	1.56	1.13	2.17
Dysregulation	0.09	0.165	1.09	0.96	1.23	0.09	0.135	1.10	0.97	1.23
Self-efficacy	0.10	0.208	1.11	0.94	1.30	0.10	0.209	1.11	0.95	1.29
Affective symptoms*	0.26	0.048	1.29	1.00	1.67	0.30	0.017	1.34	1.05	1.72
Self-harm	0.05	0.525	1.05	0.91	1.21	0.07	0.342	1.07	0.93	1.22
Anxiety	0.14	0.055	1.15	1.00	1.34	0.14	0.057	1.15	1.00	1.32
Illicit drug	0.07	0.522	1.08	0.86	1.34	0.09	0.403	1.09	0.89	1.34
Gender*	0.31	< 0.001	1.36	1.16	1.59	0.30	< 0.001	1.35	1.17	1.57
Age*	− 0.11	0.008	0.89	0.82	0.97	− 0.10	0.001	0.90	0.85	0.96
Distressed voice-hearing										
Experience of bullying*	0.22	0.005	1.24	1.07	1.45	0.20	0.007	1.23	1.06	1.42
Experience of trauma*	0.10	0.004	1.11	1.03	1.19	0.10	0.004	1.10	1.03	1.18
School absence (days)	0.00	0.741	1.00	0.98	1.02	0.00	0.687	1.00	0.98	1.02
School grades	0.01	0.902	1.01	0.87	1.17	−0.04	0.594	0.96	0.83	1.11
Negative self-worth*	1.06	<0.001	2.89	1.83	4.57	0.98	<0.001	2.66	1.73	4.10
Family support*	− 0.32	0.006	0.73	0.58	0.91	− 0.30	0.007	0.74	0.60	0.92
Distractibility*	0.47	0.002	1.60	1.18	2.17	0.42	0.004	1.53	1.14	2.04
Social dysfunction	0.35	0.182	1.42	0.85	2.37	0.34	0.172	1.41	0.86	2.31
Dysregulation*	0.22	0.024	1.25	1.03	1.51	0.25	0.007	1.29	1.07	1.54
Self-efficacy*	0.41	0.001	1.50	1.17	1.92	0.37	0.002	1.45	1.15	1.83
Affective symptoms	− 0.14	0.470	0.87	0.58	1.28	− 0.07	0.697	0.93	0.64	1.35
Self-harm*	0.26	0.004	1.29	1.08	1.54	0.26	0.002	1.30	1.10	1.53
Anxiety*	0.57	< 0.001	1.76	1.47	2.11	0.57	< 0.001	1.77	1.49	2.10
Illicit drug	0.14	0.397	1.15	0.84	1.57	0.20	0.178	1.22	0.91	1.63
Gender	0.19	0.125	1.21	0.95	1.56	0.17	0.163	1.18	0.93	1.50
Age*	− 0.16	0.021	0.86	0.75	0.98	− 0.11	0.017	0.90	0.82	0.98
*R* ^2a^	0.08	0.12				0.08	0.12			

Results for the age restricted sample (16–19 years old) and total sample (16–25 years old) are given separately

*p < 0.05

^a^Nagelkerke/Cox and Snell

Some variables were related to the category “VH” in contrast to “no VH”. These were Experience of trauma, School grades, Distractibility, Social dysfunction, Affective symptoms, Gender, and Age. Including a broader age span resulted in almost identical estimates as the model based on subjects 16–19 years old. This means that not distressing VH was more plausible among subjects with trauma experiences, higher mean academic characters, more problems regarding distractibility and social functioning, higher levels of affective symptoms, younger age and among boys in contrast to girls. The strongest statistical relation was Social dysfunction with odds ratio (OR) at 2.21, which means 121% increased risk for VH for each increase in this variable.

Regarding distressing VH, the following predictors were found to be statistically significant: experience of bullying, trauma exposure, negative self-worth, less family support, stronger levels of distractibility, dysregulation of activation, negative self-efficacy, self-harm, anxiety, and lower age. The strongest risk factor was found to be negative self-worth.

## Conclusion

Hearing thoughts out aloud without being disturbed by them versus being distressed by VH was mainly predicted by different constellations of psychosocial characteristics, suggesting that VH with and without distress represent two different groups of adolescents. Overall, this supports the emphasis put on distress in clinical practice. Not being disturbed by the voices was primarily related to social dysfunction, in addition to the experience of trauma, distractibility, affective symptoms, higher school grades, male gender and older age. Perceiving the voices as disturbing was related to the experience of bullying and trauma, negative self-worth, and self-efficacy, less family support, dysregulation of activation, distractibility, self-harm, anxiety and younger age. Negative self-worth was the strongest predictor.

As expected, the adolescents with disturbing VH were characterized by most of the typically depicted comorbid mental health symptoms in psychosis (anxiety, dysregulation of activation, self-harm), risk factors for psychosis (experience of trauma and bullying), predictors of psychosis conversion and markers of mental distress (distractibility) and fewer psychosocial moderators and resilience factors (family support, self-efficacy, experience of self-worth). Some psychosis-related psychosocial variables were not, however, related to distressing voices in this population-based sample, i.e. worse school grades, more school absence, reduced social functioning, illicit substance use and more affective symptoms. This may be explained by characteristics of the sample, as most attended high school. Adolescents with major issues related to these variables may not be part of the present sample because they probably have dropped out of school.

Adolescents not distressed by voices displayed a somewhat different profile of psychosocial characteristics as compared to their no VH peers. Social dysfunction was the strongest predictor, followed by better grades and male gender. Distractibility was related to voice-hearing in both groups, and this is consistent with several studies finding reduced attentional control in all voice hearers regardless of diagnosis and treatment needs [86]. In addition, the importance of experience of trauma was seen for both groups, in line with studies showing childhood trauma in broader groups than psychosis only [41]. The effect of lower age yielding more VH seen in previous studies [6] was also seen in both groups. The relationship to better grades for the not distressed by voices group seems paradoxical. It can be speculated that this may reflect high-or over-achieving youngsters with suboptimal healthy work-rest balance, social life and mental overload. Youngsters within a high-functioning autism/Asperger spectrum continuum with low social functioning, male gender, better grades and voice hearing could also belong in this group. Increased school related stress and a more competitive school environment have been suggested as factors for explaining increases in student mental health problems in Norway, supporting the first speculation, and in line with a somewhat increased affective symptom load.

The difference between the distressed and non-distressed group seems to be related to negative self-apprehensions, less support from family and possibly peers via bullying, all together potentially leading to reduced ability to use psychological and social coping strategies. Dysregulation of activation including questions on restlessness, anxiety and self-harm creates a picture of youngsters in emotional distress. The relationship between these variables and the development of psychosis should be studied in more detail. The present study does not test causality; whether these characteristics are an effect of the troubling voices or vice versa. The topic clearly awaits longitudinal studies.

Other questions also remain unanswered, representing limitations of the present study. We do not know whether being distressed or disturbed by the voices actually is a sign of a HR or a psychotic state, which is dependent on a more comprehensive understanding of the individual interpretations of the voices than is possible via a web-based questionnaire. For instance, there may be a few individuals within the autism-spectrum in the present sample (0.45% broader defined Autism Spectrum Disorders; [82]) who may have very concrete interpretations of “voice speaking my thoughts aloud”, but since these youngsters also have a vulnerability for psychosis exclusion would create a biased sample. The current paradigm was not set out to test whether the voice hearing was part of other mental health disorders with frequent voice hearing. Some of the risk factors reported in the present study clearly overlap with other disorder, such as borderline and dissociative disorders. We do not know what the phenomenon of hearing voices out loud actually stands for, and we do not have data on the frequency or content of these experiences, variables which have been shown to identify voice-hearers in treatment [16]. When adolescents actually seek help for their voices, they present with a high level of suffering, a reduced level of functioning and severe and/or comorbid psychopathology [87].

There is a trade-off between the comprehensiveness of topics covered by the web-based questionnaire, the sample size and the in-depth understanding of the different phenomena in question. Finally, the study did not test the relative importance of the selected features in explaining the hearing voices categories, and whether the distress-related distinction is categorical or continuous. The strengths of the present study are the high number of participants and the representativeness of the sample via the population-based inclusion [67], supported by the high rate (93%) of 16–18 year olds that attend high-school in Norway [88]. In addition, the sample size enables the possibility of examining several important variables simultaneously with high levels of statistical power.

In conclusion, the findings support the validity of the clinical distinction between adolescents being disturbed by VH or not as seen in high quality early intervention services. Since VH in itself is a relatively frequent experience in adolescence, examining the amount of personal distress related to these alternative perceptual experiences may aid referral practices and treatment decisions in services for psychosis or high risk for psychosis.
